# Silencing LINC00663 inhibits inflammation and angiogenesis through downregulation of NR2F1 via EBF1 in bladder cancer

**DOI:** 10.1080/15476286.2024.2368304

**Published:** 2024-06-18

**Authors:** Xiulong Zhong, Lijiang Sun, Junxiang Liu, Xiaokun Yang, Minghui Hou, Xinning Wang, Huifeng Diao

**Affiliations:** a Department of Urology Surgery, Affiliated Hospital of Qingdao University, Qingdao, Shandong, P.R. China; b Medical Record Management Center, Affiliated Hospital of Qingdao University, Qingdao, Shandong, P.R. China

**Keywords:** LINC00663, EBF1, nuclear receptor subfamily 2 group F member 1, bladder cancer, inflammation, angiogenesis

## Abstract

This study is to elucidate the effect of the LINC00663/EBF1/NR2F1 axis on inflammation and angiogenesis in bladder cancer (BC) and related molecular mechanisms. After transfection, functional experiments were conducted to test cell proliferation and invasion, tube formation ability, and content of inflammatory factors, Snail, E-cadherin, and VEGFA. Meanwhile, the relationships among LINC00663, EBF1, and NR2F1 were predicted and verified. In addition, xenograft experiments in nude mice were performed to observe the oncogenicity of 5637 BC cells *in vivo*. In BC tissues and cells, LINC00663 and NR2F1 were upregulated. Silencing NR2F1 or LINC00663 repressed cell proliferation and invasion, weakened vascular mimicry *in vitro*, decreased inflammatory factor, Snail, and VEGFA levels, and increased expression of E-cadherin. LINC00663 positively regulated NR2F1 expression through EBF1. Additionally, *in vivo* experiments showed that NR2F1 upregulation reversed the suppression effects of LINC00663 silencing on tumour growth, inflammation, and angiogenesis. Silencing LINC00663 decreased NR2F1 expression by mediating EBF1, thereby inhibiting BC inflammation and angiogenesis.

## 1. Introduction

Bladder cancer (BC), among the most ordinarily diagnosed malignancies, could be a major contributor to cancer-associated deaths among males, with about 549,000 newly diagnosed patients and 200,000 patients dying in 2018 [1]. Generally, BC is classified into non-muscle and muscle invasive types, whereas the former occurs in 75% of BC patients [2]. Importantly, smoking, occupational exposure, and Schistosoma haematobium infection are all regarded as risk factors for BC [3,4]. Moreover, inflammation has been shown to activate the angiogenesis and tumour progression in BC patients [5]. Surgery and chemotherapy are the predominant treatment choices for BC; however, the treatment efficacy is far from satisfactory [6]. Therefore, searching for effective targets such as tumour biomarkers is essential for predicting prognosis and guiding treatment.

Nuclear Receptor subfamily 2 group F member 1 (NR2F1) is mainly associated with tumour cell dormancy, invasion, and metastasis during cancer development and growth [7]. Reportedly, upregulated NR2F1 expression was significantly linked with the recurrence and metastasis of salivary adenoid cystic carcinoma [8]. NR2F1 was identified to be a differentially expressed gene (DEG) in BC patients associated with prognosis [9]. Nevertheless, the role and regulatory mechanism of NR2F1 in BC have not been unveiled. From LncMAP website, we found that LINC00663 may participate in BC development by binding the transcription factor EBF1 to regulate NR2F1 expression.

LncRNAs have over 200 nucleotides [10] and regulate many cellular processes (like proliferation, differentiation, invasion, metastasis, and apoptosis) through the regulation of gene expression [11]. Increasing studies showed that some lncRNAs can facilitate BC cell dissemination. For example, in patients with early-stage BC, upregulated lncRNA CASC11 expression was detected and was found to promote cancer cell proliferation by modulating its downstream gene in BC [12]. Another study displayed that exosomal lncRNA LNMAT2 was overexpressed in BC tissues and promoted lymphangiogenesis and lymphatic metastasis in BC [13]. More importantly, LINC00663 was highly expressed in pancreatic cancer and served as a prognostic biomarker of pancreatic cancer [14]. However, there is no data investigating the biological role of LINC00663 in BC. JASPAR predicted three binding sites between EBF1 and NR2F1. EBF1, a transcription factor, was verified to be involved in the transcriptional regulation of several survival-associated hub immune genes in BC [15]. Accordingly, we assumed that LINC00663 may facilitate the inflammation and angiogenesis of BC by regulating NR2F1 via EBF1, hoping to provide valuable references for developing new molecular targets for the treatment of BC.

## 2. Materials and methods

### 2.1. Bioinformatics analysis

First, the HTSeq-FPKM and survival data of BC were obtained from the University of California Santa Cruz (UCSC) database (http://xena.ucsc.edu/), and DEGs in the commencement and development of BC were preliminarily analysed by R package. The univariate Cox analysis of survival data was performed to screen out the genes associated with BC prognosis. Next, a unified and standardized pan-cancer dataset TCGA-TARGET-GTEx (PANCAN, *N* = 19131, G = 60499) was downloaded from the UCSC database (https://xenabrowser.net/). Subsequently, we obtained the expression pattern of ENSG00000175745 (NR2F1) in samples from different sources, such as primary tumour, the primary blood derived cancer-peripheral blood cohort (TCGA-LAML), and metastatic, primary blood derived cancer-bone marrow, primary solid tumour, and recurrent blood derived cancer-bone marrow cohorts of TCGA-SKCM. Additionally, we obtained a high-quality prognosis dataset from the Cancer Genome Atlas (TCGA) prognostic study [16]. Also, TARGET follow-up data were downloaded from the UCSC database (https://xenabrowser.net/datapages/). Furthermore, log2(x + 0.001) transformation was conducted for each expression value. Finally, cancer types with <10 samples in one cancer type were excluded, and the expression profiles of 44 cancer types and the overall survival data were collected. Next, the coxph function of R survival package 3.2-7 was used to establish the Cox proportional hazards regression model and analyse the relationship between gene expression and prognosis. Statistical tests were performed to obtain prognostic significance using the Logrank test.

### 2.2. Sample collection

BC tissues and normal para-cancer tissues were excised from 30 BC patients in the Urology Department of Affiliated Hospital of Qingdao University from February 2019 to February 2021. Their pathological features are summarized in Table 1. The collected cancer tissues were reconfirmed by two experienced pathologists. Each participant was pathologically diagnosed with BC, and none received chemotherapy, radiotherapy, or other tumour-related treatments before the operation or suffered from other malignant tumours or bladder diseases. The collected tissue samples were immediately frozen in liquid nitrogen and then preserved at −80°C for subsequent experiments.

**Table 1.** The clinical data of patients with bladder cancer.

Pathological features	Cases
Gender	
Male	25
Female	5
Age	
≤60	14
>60	16
TNM stage	
I	10
II	8
III	8
IV	4
Lymph node metastasis	
no	20
yes	10

TNM, tumour-node-metastasis.

This study was approved by the Ethics Committee of Affiliated Hospital of Qingdao University (approval number: QYFY WZLL 28,112) and complied with the *Declaration of Helsinki*, and all patients had signed written informed consent.

### 2.3. Cell culture

BC cell lines (HT-1376 [iCell-h077], 5637 [iCell-h232], J82 [iCell-h115], and T24 [iCell-h208]), human normal bladder epithelial cells (SV-HUC-1; iCell-h199), and human umbilical vein endothelial cells (HUVECs; iCell-h110) were provided and validated by iCell Bioscience Inc. (Shanghai, China). Cells were cultivated in Roswell Park Memorial Institute (RPMI)-1640 medium (Gibco, Grand Island, NY, USA), which contained 10% foetal bovine serum (FBS; Thermo Fisher Scientific, Wilmington, DE, USA) and penicillin–streptomycin (Gibco) at 37°C with 5% CO_2_.

### 2.4. Cell transfection

NR2F1 knockdown vector (sh-NR2F1) (sequence: GGCAGCGATCTCCATAGAAAC), LINC00663 knockdown vector (sh-LINC00663) (sequence: CCTGACATCATAATGGTAGAT), EBF1 overexpression vector (oe-EBF1), NR2F1 overexpression vector (oe-NR2F1), and their negative controls (NCs) were procured from Hanbio Biotechnology (Shanghai, China). The transfection titre of lentiviral vector was 1 × 10^8^ TU/mL. Follow-up assays were performed after 48-h transfection.

### 2.5. Quantitative reverse transcription polymerase chain reaction (qRT-PCR)

The total RNA was extracted and reverse-transcribed with TRIZOL (Invitrogen, Carlsbad, CA, USA) and the RT kit (Takara, Tokyo, Japan). Gene expression was measured using SYBR Green Mix (Takara) on a Biosystems 7300 system (ABI, Foster City, CA, USA). Three replicates were set for each reaction. Data were analysed using the 2^−ΔΔCt^ method [17] (internal reference: glyceraldehyde-3-phosphate dehydrogenase [GAPDH]). Related primers are listed as follows: LINC00663 (F: 5′-GCTTGTAGCCCCTTTCTTTTGG-3′, R: 5′-AGTCCCTTCTGCCTATGACCCT-3′), EBF1 (F: 5′-TGCAGATCTGGTTGAAGCCCTGTA-3′, R: 5′-ATCCCTGCATGGACCGAAGTGTT-3′), NR2F1 (F: 5′-ATCCGAGCTACAAAGCATGG-3′, R: 5′-TCCACAT CCGTCCACAATAA-3′), and GAPDH (F: 5′-CACCCACTCCTCCACCTTTG-3′, R: 5′-CCACCACCCTGTTGCTGTAG-3′).

### 2.6. Western blot

Cells or tissues were lysed by radio-immunoprecipitation assay (RIPA) lysis (Beyotime, Shanghai, China) on ice for 15 min and centrifuged (13000 g, 5 min), and then protein concentration was assessed on a bicinchoninic acid (BCA) kit (Beyotime). The protein was denatured in boiling water for 10 min after the loading buffer was added and the loading volume was calculated based on protein loaded. After that, the protein underwent electrophoresis (30 min at 80 V, 90 min at 120 V) and then transferred onto a polyvinylidene fluoride (PVDF) membrane at 250 mA for 100 min, followed by thrice of membrane rinsing, each for 1–2 min. Afterwards, the membrane was treated with a blocking buffer for 2 h and incubated overnight (4°C) with primary antibodies of NR2F1 (1:1000, ab181137, Abcam, Cambridge, UK), EBF1 (1:1000, sc -137,065, Santa Cruz, CA, USA), VEGFA (1:1000, ab1316, Abcam), Snail (1:1000, ab216347, Abcam), E-cadherin (1:1000, ab1416, Abcam), and GAPDH (1:2000, ab9485, Abcam). Subsequently, the membrane was washed thrice using Tris-buffered saline-tween (TBST) and probed with goat anti-rabbit immunoglobulin G (IgG) (A0208, 1:1000, Beyotime) for 2 h. Following the addition of enhanced chemiluminescence developing solution (ECL, P0018FS, Beyotime), the membrane was imaged on a chemiluminescence imaging system (Bio-Rad, Hercules, CA, USA) and analysed using Quantity One v4.6.2 software.

### 2.7. RNA-FISH

FISH assay was conducted to identify the subcellular localization of LINC00663 in BC cells. Briefly, the cells seeded in 24-well plates (6 × 10^4^ cells/well) were incubated overnight with 250 μL buffer and 250 μL FISH probes (300 ng/mL; Biosense, Guangzhou, China) in a hybridization buffer at 42°C. Air-dried slides were mounted with 4,6-diamidino-2-phenylindole. Finally, images in five different fields were obtained and examined with a fluorescence microscope (Olympus IX51, Japan).

### 2.8. Cell counting kit-8 (CCK-8) assay

After transfection, the cells were seeded onto 96-well plates. Then 10 µL of CCK-8 reagent was added to each well for 0, 24, 48, and 72 h, respectively. On a microplate reader (model 680; Bio-Rad), cell absorbance was tested at 450 nm from three independent tests.

### 2.9. Colony-forming assay

Transfected cells were digested by trypsin, centrifuged (25°C, 1500 rpm) for 5 min, and then resuspended in a complete medium. A 6-well plate was covered with 2 mL complete medium (37°C), and 500 cells were seeded onto each well for 2–3 weeks (37°C, 5% CO_2_). When cell colonies were visible to naked eyes, the cell culture was terminated. After the culture medium was absorbed, the plate was washed twice with PBS before methanol (1.5 mL) was added to fix cells for 15 min. After methanol removal, Giemsa (1 mL) was added for 20-min staining in darkness. Later, the Giemsa was washed away, and the 6-well plate was inverted on clean absorbent paper. The plate was dried, and cloned cells were counted.

### 2.10. Tube formation experiment

Matrigel (Corning, Tewksbury, MA, USA) was dissolved at 4°C, and a 96-well plate (Millipore, Billerica, MA, USA) and a pipette were pre-chilled. The plate added with 100 μL Matrigel was put in an incubator (37°C, 30 min) to solidify the Matrigel. HUVECs were re-suspended in a conditioned medium of 5637 cells and seeded onto the 96-well plate (2 × 10^4^ cells/well) with three replicate wells, followed by 5-h incubation (37°C, 5% CO_2_). Under three fields, the image was collected through the microscope with the number and length of the branches measured and recorded.

### 2.11. Transwell assay

Following cells (1 × 10^5^) were suspended in a serum-free DMEM, the upper chamber (coated with Matrigel) was added with the cell suspension, and the lower chamber was filled with 600 µL medium (containing 10% FBS). After 48 h, the cells attached to the membrane underwent fixation by 100% methanol and staining by 0.1% crystal violet, while the non-invaded cells were removed with cotton swabs. The invaded cell numbers were counted under a microscope (200 ×).

### 2.12. Enzyme-linked immunosorbent assay (ELISA)

The samples were added to the 96-well ELISA plate of the ELISA kits (R&D, Systems, UK) for overnight, followed by PBS washing (3 × 5 min) after culture solution was discarded. Later, the plate was added with 5% BSA blocking liquid (100 μL/well) for 1 h and then PBS (containing 5% BSA)-diluted primary antibody for 3 h. After PBS washing, the plate was appended with HRP-labelled secondary antibody (diluted by PBS). Finally, 10 μL of substrate was added to the supernatant or serum (37°C, 10–15 min), and the absorbance value at 450 nm was assessed.

### 2.13. Dual-luciferase assay

Target genes were screened through bioinformatics prediction. Based on the predicted results, wild and mutated promoter sequences (wt-NR2F1 and mut-NR2F1) were designed, synthesized, and inserted into pGL3-Basic vectors, followed by co-transfection into BC cells with sh-LINC00663 and sh-NC or oe-EBF1 and oe-NC (transfection titre of lentiviral vector: 1 × 10^8^ TU/mL). After 48 h, cells were lysed for the measurement of luciferase activity with a luciferase detection kit (K801–200, Biovision, Mountain View, CA, USA) and luciferase analysis system (Promega, Madison, WI, USA) (internal reference: Renilla luciferase).

### 2.14. RNA immunoprecipitation (RIP) assay

Subsequent to PBS washing and centrifugation (1500 rpm, 5 min), cells were completely mixed with RIP lysis (an equal volume). The magnetic beads were resuspended in 100 μL RIP wash buffer and added with 5 µg anti-EBF1 (sc -137,065, 1:100, Santa Cruz) for 30 min (IgG as control). Next, RIP wash buffer (500 µL) was used to wash the bead–antibody complex twice. After supernatant removal, the complex was mixed with 500 µL wash buffer. Then the tube was put on the magnetic rack, the supernatant was removed, and RIP buffer (900 µL) was appended. Subsequently, the prepared cell lysate was rapidly thawed and centrifuged (14000 rpm, 10 min, 4°C) to obtain 100 µL supernatant, which was incubated with the bead–antibody complex (4°C, overnight). Subsequent to transient centrifugation and supernatant removal, the complex was treated with RIP wash buffer (500 µL) six times. After that, Proteinase K buffer (150 µL) was used to purify the RNA by incubating (55°C, 30 min) with the bead–antibody–RNA complex. After RNA extraction, LINC00663 expression was assessed by qRT-PCR.

### 2.15. Chromatin immunoprecipitation (ChIP)

Cells were fixed with formaldehyde for 10 min and then treated with an ultrasonic cell disruptor (15 cycles for 10 s each time with intervals of 10 s) to break chromatin into fragments. After that, centrifugation was performed at 12,000 g for 10 min at 4°C to collect the supernatant, which was packaged into two tubes. Next, IgG antibody (ab172730, 1:100, Abcam) and EBF1 antibody (sc -137,065, 1:100, Santa Cruz) were added to the supernatant for overnight incubation at 4°C. The DNA–protein complex was precipitated by Protein Agarose/Sepharose, followed by 5-min centrifugation at 12,000 g and removal of supernatant. The non-specific complex was rinsed, and the bead–DNA–protein complexes were de-crosslinked (65°C, overnight). The purified DNA products were measured by qPCR to detect the binding of EBF1 to NR2F1 using NR2F1-specific primers. Each experiment was run in triplicate.

### 2.16. Animal experiment

Animal experiments were approved by the Ethics Committee of Affiliated Hospital of Qingdao University. Male BALB/c nude mice (*n* = 24, 4–6 weeks, 19 ± 2 g) from Shanghai Laboratory Animal Center were reared in specific pathogen-free animal rooms at 25–27°C and 45–50% humidity. These nude mice were randomized into four groups with six mice per group (Control, sh-NC + oe-NC, sh-LINC00663 + oe-NC, and sh-LINC00663 + oe-NR2F1 groups). According to grouping, transfected 5637 cells were collected into centrifuge tubes (10 mL) for centrifugation with the supernatant discarded. After normal saline washing, the cells were digested by 0.5% trypsin (1 mL) in a 37°C incubator, followed by preparation of single-cell suspension by blowing evenly. Later, the cells were transferred to centrifuge tubes (15 mL) and centrifuged (5 min, 300 g) with the supernatant removed, followed by twice washing in PBS. After an appropriate amount of PBS was added, the cells were counted. Next, cells (5 × 10^6^) were resuspended in 50 µL normal saline and then mixed with 50 µL Matrigel. Nude mice were anesthetized by 60 mg/kg sodium pentobarbital (i.p.). After that, the left inguinal skin was sterilized and subcutaneously inoculated with 5637 cells. Afterwards, tumour volume was recorded every 7 days based on the formula: V (mm^3^) = (A^2^ × B)/2 (A: the long diameter. B: the short diameter). Five weeks later, all mice were euthanized by cervical dislocation, and subcutaneous tumours were isolated and weighted for follow-up experiments.

### 2.17. Immunohistochemistry (IHC)

Tumours isolated from mice were immersed in 4% paraformaldehyde for 48 h of fixation and prepared into paraffin sections (4 μm thick). The sections were baked for 20 min, deparaffined with conventional xylene, dehydrated, and washed once with distilled water and thrice with PBS. After blocked with 3% H_2_O_2_ for 10 min and PBS washing, sections were subjected to heat-induced antigen retrieval. After PBS washing again, the sections were soaked in a normal goat serum blocking buffer for 20 min. Later, the sections were incubated with the primary antibodies against Ki67 (ab15580, Abcam) and CD34 (ab81289, Abcam) at 4°C overnight and then the secondary antibody for 1 h. Following washing, the sections were stained with diaminobenzidine for 1–3 min, followed by 3 min of haematoxylin staining, dehydration, clearing, and sealing. Three fields were selected for observation under a microscope (×200) and analysed using the Image J software. Next, two experienced pathologists scored the percentage of positive cells (0, <5%; 1, 5~25%; 2, 26~50%; 3, 51~75%; and 4, 76~100%) and staining intensity (0, colourless; 1, light yellow; 2, brown-yellow; and 3, dark brown) in a double-blind fashion using semi-quantitative results. The product of the two scores was the positive grade: 0, negative; 1 ~ 4, weakly positive; 5 ~ 8, positive; and 9 ~ 12, strongly positive. Finally, the microvessel density (MVD) was calculated. The MVD count was calculated based on the CD34 positive cells, clusters or individual endothelial cells, or cells with clear boundaries with adjacent tumour cells, microtubules and their surrounding connective tissues. If the structure was not connected, it was regarded as a tube [18].

### 2.18. Statistical analysis

Data were analysed using GraphPad Prism 7.0 and depicted as mean ± standard deviation. The *t*-test was employed for comparisons between two groups and one-way analysis of variance for those among multiple groups. Post hoc analysis was done with Tukey′s multiple comparison test. *p* < 0.05 was indicative of statistical significance.

## 3. Results

### 3.1. NR2F1 was overexpressed in BC tissues

**Figure 1.**
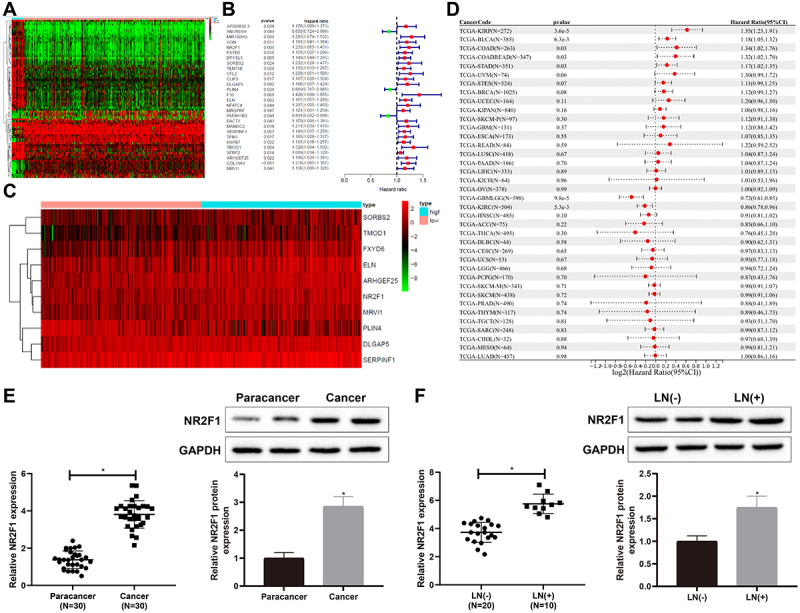
NR2F1 expressed at a high level in BC tissues.

First, we found 80 DEGs involved in the progression of BC through bioinformatics prediction (Figure 1A). Through the analysis of survival data by univariate Cox analysis, it was found that 28 DEGs were related to BC prognosis (Figure 1B). Nevertheless, through multivariate Cox analysis on the 28 DEGs, 10 genes were found to be linked with BC poor prognosis (Figure 1C, Supplementary table S1). Combining the results from univariate and multivariate Cox analyses, we focused on NR2F1, a gene associated with poor prognosis of BC. Subsequently, we analysed the relationship between NR2F1 and BC prognosis using pan-cancer dataset downloaded from the UCSC database and prognostic dataset from the TCGA dataset in a previous study [16]. The results demonstrated that the elevated NR2F1 in BC predicted poor prognosis, indicating that NR2F1 was closely related to BC progression (Figure 1D, Supplementary table S2).

(A) bioinformatics prediction was used to analyse the DEGs in the occurrence and development of BC. (B) univariate Cox analysis on survival data was used to analyse the genes related to BC prognosis. (C) multivariate Cox analysis was used to analyse the genes related to BC prognosis. (D) the relationship between NR2F1 and BC prognosis was analysed using a pan-cancer dataset downloaded from the UCSC database and a prognostic dataset from the TCGA database in a previous study. (E) the expression of NR2F1 in BC tissues and normal para-cancer tissues was measured by qRT-PCR and western blot. (F) the expression of NR2F1 in tissues of patients with/without lymph node metastasis was detected by qRT-PCR and western blot. *p < 0.05, compared with para-cancer, or LN(-) group. BC, bladder cancer; DEGs, differentially expressed genes; LN, lymph node metastasis.

Next, qRT-PCR and western blot revealed that NR2F1 was increased in BC tissues versus normal para-cancer tissues (Figure 1E, *p < 0.05). Meanwhile, compared with BC patients without lymph node metastasis, NR2F1 was signally upregulated in BC patients with lymph node metastasis (Figure 1F, *p < 0.05). Overall, NR2F1 was upregulated in BC tissues.

### 3.2. Knockdown of NR2F1 inhibited inflammation and vascular mimicry in BC cells

**Figure 2.**
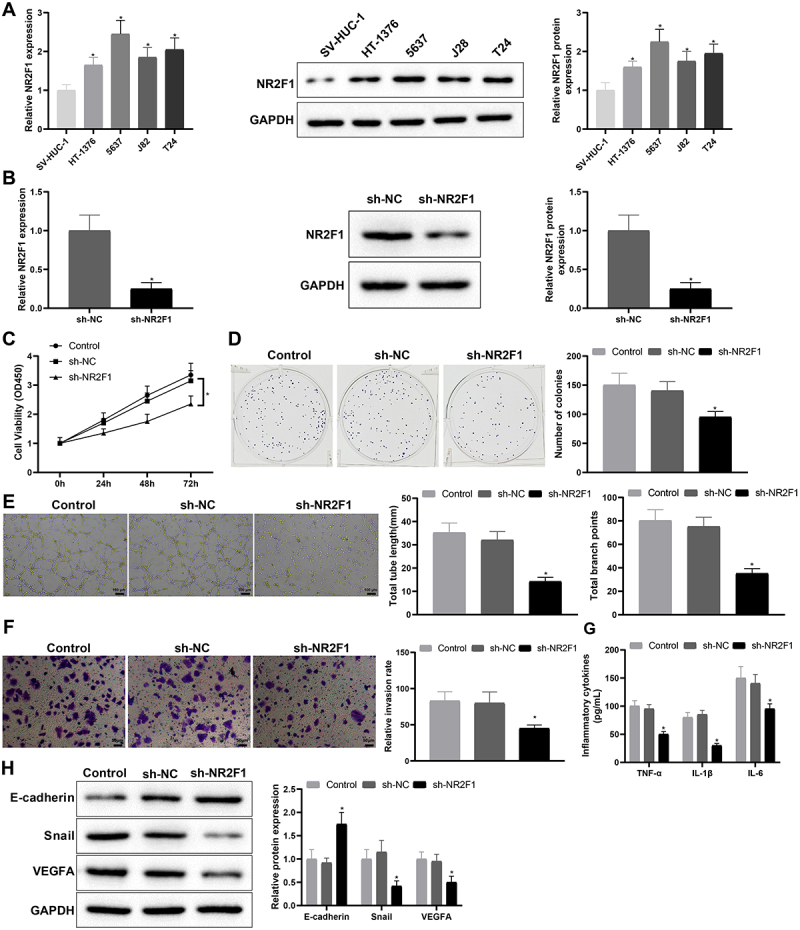
Low expression of NR2F1 suppressed inflammation and vascular mimicry in BC cells.

Compared with SV-HUC-1 cells, NR2F1 was conspicuously elevated in HT-1376, 5637, J82, and T24 cells, and the elevation was relatively significant in 5637 cells (Figure 2A, *p < 0.05). Subsequently, we constructed the NR2F1 knockdown vector, and screened and verified the transfection efficiency of the NR2F1 knockdown vector by qRT-PCR, which manifested that the knockdown efficiency of sh-NR2F1-1 was the most significant (Supplementary Figure S1A). Hence, the subsequent experiments used sh-NR2F1-1 (hereinafter referred to as sh-NR2F1) for functional experiments. Following sh-NR2F1 or sh-NC transfection, qRT-PCR and western blot measurements of transfection efficiency showed that the sh-NR2F1 group had obviously lower NR2F1 expression than the sh-NC group (Figure 2B, *p < 0.05). As suggested by CCK-8 and colony-forming assays, the sh-NR2F1 group had decreased cell proliferation (Figure 2C–D, *p < 0.05). Tube formation experiments suggested that the sh-NR2F1 group had shorter length of newly formed pseudo-tubules and decreased number of newly formed nodes (Figure 2E, *p < 0.05), demonstrating a weakened ability of vascular mimicry in response to NR2F1 suppression. Results from the transwell assay showed that the transfection of sh-NR2F1 markedly repressed cell invasion ability (Figure 2F, *p < 0.05). ELISA showed that TNF-α, IL-6, and IL-1β levels were reduced in the sh-NR2F1 group (Figure 2G, *p < 0.05). Western blot analysis reflected that the sh-NR2F1 group had increased E-cadherin expression but decreased expression of Snail and VEGFA (Figure 2H, *p < 0.05). Conclusively, silencing NR2F1 may suppress inflammation and angiogenesis in BC.

(A) the expression of NR2F1 in BC cells (HT-1376, 5637, J82, and T24) and human normal bladder epithelium (SV-HUC-1) were assessed by qRT-PCR and western blot. (B) the transfection efficiency was measured by qRT-PCR and western blot. (C-D) CCK-8 and colony-forming assays were used to test cell proliferation. (E) the ability of vascular mimicry was evaluated through tube formation experiment. (F) cell invasion ability was tested by transwell assay. (G) the levels of TNF-α, IL-6, and IL-1β were measured by ELISA. (H) the expression of Snail, E-cadherin, and VEGFA was examined by western blot. Data were expressed as mean ± standard deviation, and each experiment was repeated three times. *p < 0.05, compared with SV-HUC-1 or sh-NC group. BC, bladder cancer.

### 3.3. LINC00663 positively regulated NR2F1 expression

**Figure 3.**
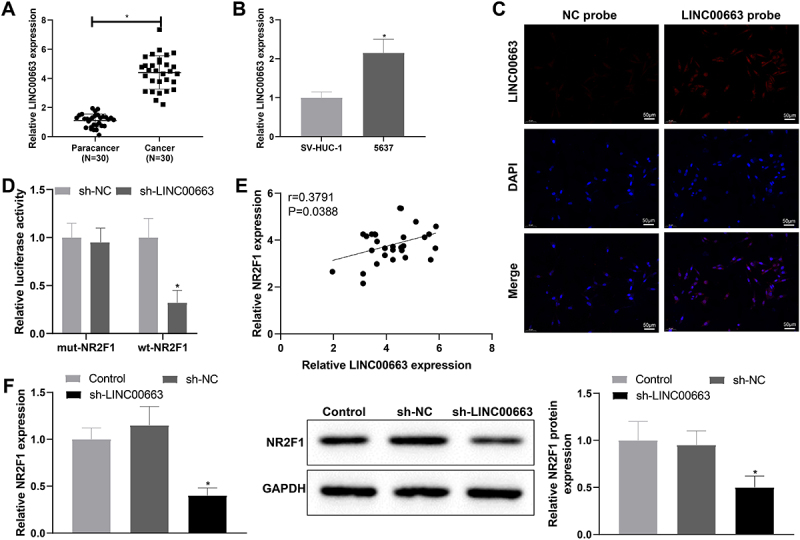
LINC00663 positively modulated NR2F1.

Based on the above findings, we further explore the relevant regulatory axis of NR2F1 through the website LncMAP (http://bio-bigdata.hrbmu.edu.cn/LncMAP/index.jsp). Results manifested that LINC00663 may be involved in BC process by binding to transcription factor EBF1 to regulate NR2F1 expression (Supplementary table S3). Furthermore, there is no evidence reporting the implication of LINC00663 in BC. Afterwards, qRT-PCR revealed that LINC00663 was strikingly increased in BC tissues and 5637 cells versus normal para-cancer tissues and SV-HUC-1 cells, respectively (Figure 3A–B, *p < 0.05). With respect to FISH assay results, LINC00663 was predominantly localized in the nuclei of BC cells (Figure 3C).

(A-B) qRT-PCR was used to measure the expression of LINC00663 in BC tissues and cells. (C) FISH assay was used to detect the localization of LINC00663 in BC cells. (D) the binding of LINC00663 and NR2F1 was verified by dual-luciferase reporter gene assay. (E) the correlation between LINC00663 and NR2F1 was analysed by Pearson. (F) after 5673 cells were transfected with sh-LINC00663, the expression of NR2F1 was evaluated by qRT-PCR and western blot. Data were expressed as mean ± standard deviation, and each cellular experiment was repeated three times. *p < 0.05, compared with para-cancer, SV-HUC-1 or sh-NC group. BC, bladder cancer.

To dissect whether there is an interaction between LINC00663 and NR2F1, we constructed three knockdown vector sequences for LINC00663, followed by qRT-PCR. As evidenced in Supplementary figure S1B, the knockdown efficiency of LINC00663-2 (hereinafter noted as sh-LINC00663) was the most significant, which was used for functional experiments in follow-up assays. Dual-luciferase assay illustrated that sh-LINC00663 downregulated the luciferase activity of wt-NR2F1 but had no effect on that of mut-NR2F1 (Figure 3D, *p < 0.05), which indicated that LINC00663 might regulate its promoter activity. In addition, through Pearson correlation analysis, NR2F1 was positively correlated with LINC00663 (Figure 3E). To further confirm the effect of LINC00663 on NR2F1 expression, we transfected 5673 cells with sh-LINC00663, in which the NR2F1 expression was detected. In comparison with the sh-NC group, NR2F1 was obviously reduced in sh-LINC00663 group (Figure 3F, *p < 0.05). Overall, LINC00663 could positively modulate the NR2F1 expression.

### 3.4. Silencing LINC00663 repressed inflammation and vascular mimicry in BC cells by regulating NR2F1 expression

**Figure 4.**
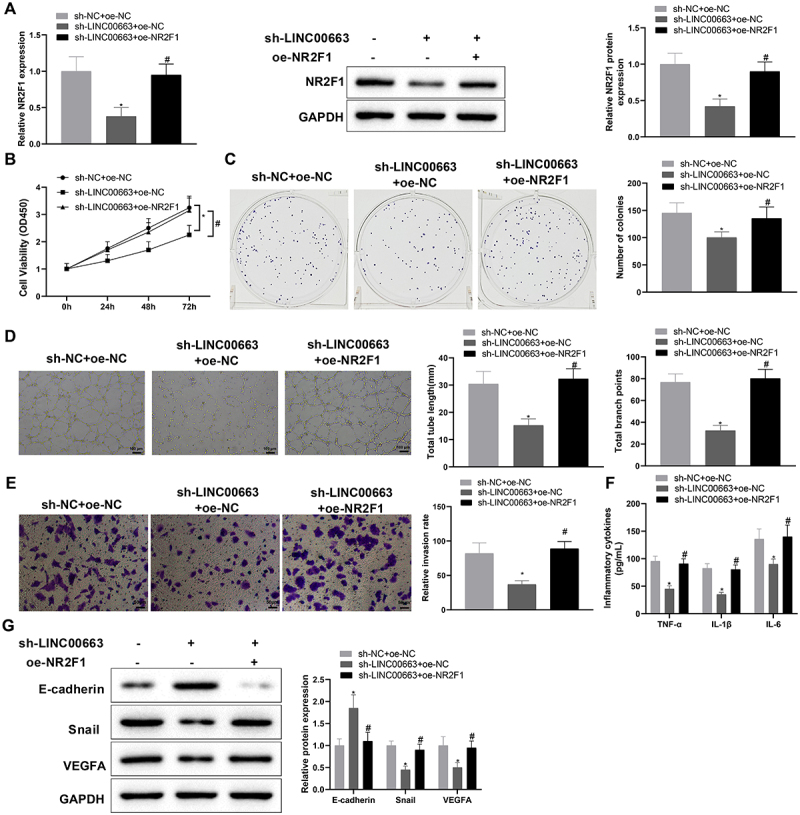
Knockdown of LINC00663 inhibited inflammation and vascular mimicry in BC cells by decreasing NR2F1 expression.

Next, 5673 cells were co-transfected with sh-LINC00663 and oe-NR2F1 to determine whether LINC00663 affects BC inflammation and angiogenesis by regulating NR2F1 expression. qRT-PCR and western blot manifested that NR2F1 expression was reduced in the sh-LINC00663 + oe-NC group (vs. sh-NC + oe-NC group) but elevated in the sh-LINC00663 + oe-NR2F1 group (vs. sh-LINC00663 + oe-NC group) (Figure 4A, *p < 0.05). Later, we conducted functional experiments to explore the effect of the LINC00663/NR2F1 regulatory axis on inflammation and vascular mimicry in BC cells. In comparison with the sh-NC + oe-NC group, the sh-LINC00663 + oe-NC group had decreased cell proliferation and invasion, reduced TNF-α, IL-6, and IL-1β levels, and weakened the ability of vascular mimicry (Figure 4B–F, *p < 0.05). Also, as reflected in Figure 4G (*p < 0.05), E-cadherin expression was increased, and Snail and VEGFA were visibly decreased in the sh-LINC00663 + oe-NC group (vs. sh-NC + oe-NC group). However, after the co-transfection of sh-LINC00663 and oe-NR2F1, upregulation of NR2F1 reversed the repressive effect of LINC00663 deficiency to promote inflammation and vascular mimicry in BC cells (Figure 4B–G, *p < 0.05). Altogether, LINC00663 knockdown may inhibit inflammation and vascular mimicry in BC cells by decreasing NR2F1 expression.

After 5637 cells were transfected with sh-LINC00663 and oe-NR2F1, (A) the transfection efficiency was tested with qRT-PCR and western blot. (B-C) cell proliferation was evaluated by CCK-8 and colony-forming assays. (D) the ability of vascular mimicry was assessed by tube formation experiments. (E) cell invasion was detected by transwell assay. (F) the levels of TNF-α, IL-6, and IL-1β were measured by ELISA. (G) the expression of Snail, E-cadherin, and VEGFA was examined by western blot. Data were shown as mean ± standard deviation, and each assay was repeated three times. *p < 0.05, compared with the sh-LINC00663 + oe-NC group; ^#^*p* < 0.05, compared with the sh-LINC00663 + oe-NC group. BC, bladder cancer.

### 3.5. LINC00663 upregulated NR2F1 expression by binding EBF1 to facilitate inflammation and vascular mimicry in BC cells

**Figure 5.**
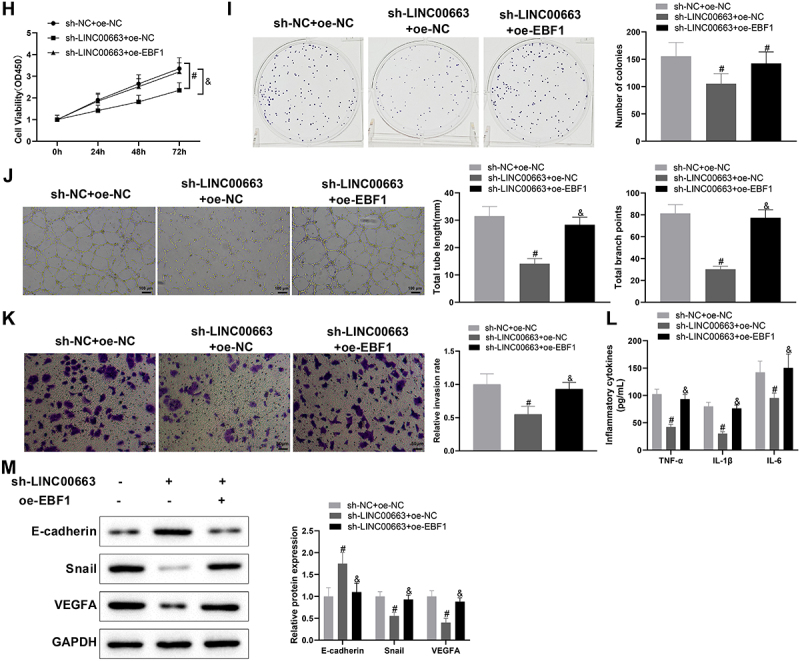
(Continued). (A) RIP assay was used to verify the binding between LINC00663 and EBF1. (B) the binding sites between EBF1 and NR2F1 were predicted by the JASPAR website. (C-E) the binding relationship between EBF1 and NR2F1 was verified by dual-luciferase reporter gene assay and ChIP assay (groups of site 1/2/3 referred to qPCR primers designed according to sequences of site 1/2/3 of NR2F1). (F) the binding between LINC00663 and NR2F1 was verified by ChIP assay. After BC cells were transfected with sh-LINC00663 and oe-EBF1, (G) the expression of NR2F1 was measured by qRT-PCR and western blot. (HI) cell proliferation was tested by CCK-8 and colony-forming assays. (J) the ability of vascular mimicry was examined by tube formation experiments. (K) cell invasion was measured by transwell assay. (L) the levels of TNF-α, IL-6, and IL-1β were measured by ELISA. (M) the expression of Snail, E-cadherin, and VEGFA was examined by western blot. Data were exhibited as mean ± standard deviation, and each experiment was run in triplicate. *p < 0.05, compared with IgG, oe-NC, or Distal group; ^#^*p* < 0.05, compared with sh-NC or sh-NC + oe-NC group; ^&^*p* < 0.05, compared with the sh-LINC00663 + oe-NC group. BC, bladder cancer.

According to the above experimental results and LncMAP analysis, we speculated that LINC00663 might mediate BC inflammation and angiogenesis by binding EBF1 to modulate NR2F1 expression. RIP assay revealed that the EBF1 group had a higher enrichment of LINC00663 (vs. IgG group) (Figure 5A, *p < 0.05), suggesting that the EBF1 protein could specifically bind to LINC00663. Subsequently, we predicted the binding sites between EBF1 and NR2F1 through the JASPAR website (http://jaspar.genereg.net/), and the results showed that there were three binding sites between them (Figure 5B). In addition, dual-luciferase assay demonstrated that site 3 (557–567 nt) was a specific site for EBF1 binding to NR2F1 promoter region (ATCTCCAGGGT) (Figure 5C–D, *p < 0.05). Next, ChIP assay displayed that, compared with the IgG group, the amplification product obtained by site 3 primer in EBF1 group was more than that obtained by Distal primer (Figure 5E, *p < 0.05), further suggesting that EBF1 bound NR2F1 DNA at site 3. Moreover, the ChIP assay unveiled that the NR2F1 promoter in sh-LINC00663 group was markedly reduced versus sh-NC group (Figure 5F, ^#^*p* < 0.05). The above findings indicated that LINC00663 regulated NR2F1 expression through EBF1.
**Figure 5.**
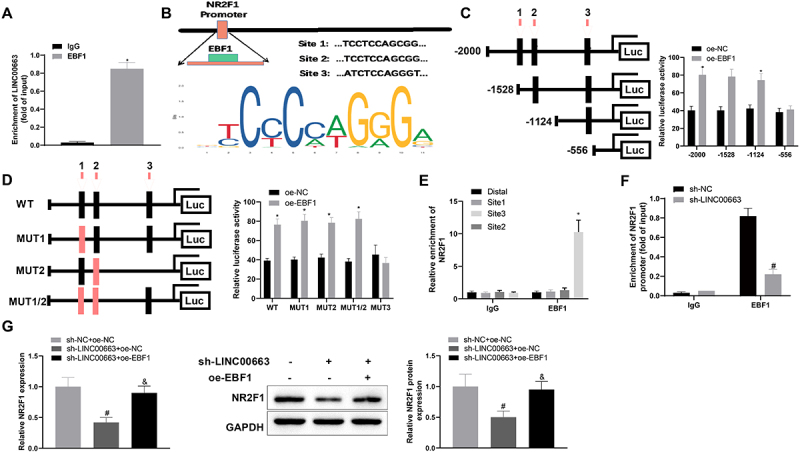
LINC00663 modulated NR2F1 expression through EBF1 and affected inflammation and vascular mimicry in BC cells.

Subsequently, BC cells were co-transfected with sh-LINC00663 and oe-EBF1. Results manifested that overexpressed EBF1 promoted NR2F1 expression (Figure 5G, ^&^*p* < 0.05). As expected, overexpressed EBF1 could facilitate inflammation and vascular mimicry in BC cells by reversing the inhibitory effect of sh-LINC00663 (Figure 5H-M, ^&^*p* < 0.05). All results suggested that LINC00663 may modulate NR2F1 expression by binding EBF1, thereby affecting BC inflammation and angiogenesis.

### 3.6. Knockdown of LINC00663 inhibited tumor growth by reducing NR2F1 expression

**Figure 6.**
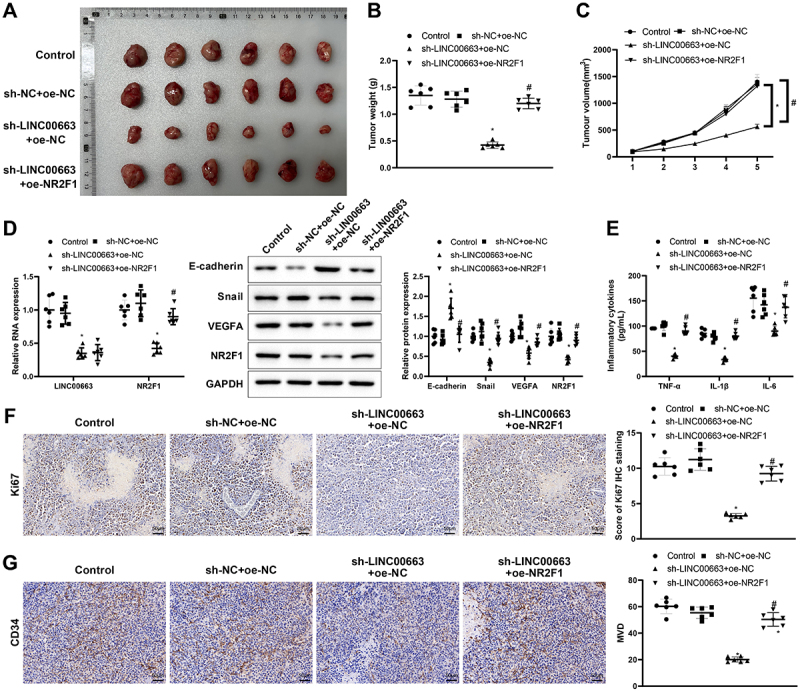
Silencing LINC00663 repressed tumour growth by reducing NR2F1 expression.

Finally, we transplanted cancer cells into nude mice to explore their manifestations *in vivo*. In comparison with the sh-NC+ oe-NC group, the tumour weight and volume were visibly decreased in the sh-LINC00663 + oe-NC group (Figure 6A–C, *p < 0.05), showing that inhibition of LINC00663 markedly repressed the tumour growth. The sh-LINC00663 + oe-NC group had decreased expression of LINC00663, NR2F1, Snail, and VEGFA, and increased expression of E-cadherin (Figure 6D, *p < 0.05). TNF-α, IL-6, and IL-1β levels were obviously reduced in the sh-LINC00663 + oe-NC group (Figure 6E, *p < 0.05). Ki67 expression and MVD were decreased in the sh-LINC00663 + oe-NC group (Figure 6F–G, *p < 0.05). However, overexpression of NR2F1 nullified the inhibitory effect of LINC00663 knockdown on tumour growth (Figure 6A–G, ^#^*p* < 0.05). In summary, silencing LINC00663 repressed tumour growth by reducing NR2F1 expression.

(A) xenograft tumour extracted from nude mice. (B) the weight of tumour was analysed.(C) the growth curve was used to evaluate the growth rate. (D) the expression of LINC00663, NR2F1, Snail, VEGFA, and E-cadherin was measured by qRT-PCR or western blot. (E) the levels of TNF-α, IL-6, and IL-1β were tested by ELISA. (F) IHC assay was used to measure the expression of Ki67. (G) IHC assay was used to assess the change of MVD. Data were expressed as mean ± standard deviation, *n* = 6. *p < 0.05, compared with sh-NC+ oe-NC group; ^#^*p* < 0.05, compared with the sh-LINC00663 + oe-NC group. MVD, microvessel density; IHC, Immunohistochemistry.

## 4. Discussion

BC is a malignancy of the urinary system with a high recurrence rate [19]. Therefore, finding effective biomarkers for BC is imperative for early diagnosis of this disease. Nowadays, the function of lncRNAs in BC pathogenesis has been emphasized by numerous studies [20]. In the present study, we first identified NR2F1 was closely related to BC prognosis through bioinformatics analysis, and then found the regulatory axis related to NR2F1 in BC. Additionally, a string of assays both *in vivo* and *in vitro* were performed to verify the role of the LINC00663/EBF1/NR2F1 axis in BC progression, and results showed that silencing LINC00663 inhibited inflammation and angiogenesis in BC partially by decreasing NR2F1 expression via binding EBF1.

Based on the bioinformatics analysis, upregulated expression of NR2F1 was associated with BC poor prognosis. Furthermore, NR2F1 expression was detected to be significantly increased in BC tissues, especially in patients with lymph node metastasis. Knockdown of NR2F1 repressed inflammation and vascular mimicry in BC cells, shown by reduced TNF-α, IL-6, IL-1β, Snail, and VEGFA levels and increased E-cadherin levels. Snail has been reported to be abnormally expressed in colorectal cancer, gastric cancer, liver cancer, lung adenocarcinoma, and other tumours; moreover, Snail up-regulation is associated with tumour invasion, metastasis, and poor prognosis [21–24]. The activation of VEGFA could promote tumour growth [25]. Down-regulated expression of E-cadherin has been found in many tumours, and the absence of E-cadherin is often associated with poor patient outcomes [26]. The inhibition of inflammatory factor levels helped to shape a non-inflamed tumour microenvironment in BC [27]. NR2F1 also assumed an important role in shaping the tumour microenvironment of BC [28], which indicated that NR2F1 is a potential biomarker for predicting the efficacy of immunotherapy for BC. In a previous study, NR2F1 was found to be involved in DNA base-pair repair, inflammation, and oncogenic pathways [29]. NR2F1 was verified to be a key modulator of neuro-inflammation in the adult hippocampus [30]. Moreover, a prior study demonstrated that NR2F2 could play an important role in tumorigenesis by regulating multiple signalling pathways and controlling tumour cell growth and angiogenesis [31]. In pancreatic cancer, increased NR2F1 resulted in promoted cancer cell biological behaviours, including proliferation, migration, and invasion [32]. Notably, NR2F1 was markedly reduced in several cancers, such as head and neck squamous cell carcinoma and breast carcinoma [33]. The discrepancy suggests that NR2F1 may have different functions depending on cancer types. Owing to the controversial role of NR2F1 in different cancers, we will further explore the related molecular mechanisms in future studies.

Next, we searched its related regulatory axis on the LncMAP website and found the axis of LINC00663/EBF1/NR2F1. LINC00663 was detected to be overexpressed, and silencing LINC00663 resulted in inhibited inflammation and angiogenesis in xenograft tumours formed by 5637 BC cells. In pancreatic cancer samples, LINC00663 expression was measured to be higher as compared to normal pancreatic samples [14]. Also, LINC00663 was reported to be highly expressed in glioma, and LINC00663 knockdown could restrain cell viability by modulating AKT/mTOR pathway [34]. The above data suggested that LINC00663 may be an oncogene in the occurrence and development of cancers. Previous researchers have emphasized the crucial roles of lncRNAs in inflammatory responses and angiogenesis of different cancers. For example, LINC00665 exerted an important pro-inflammatory role by activating the NF-κB pathway in hepatocellular carcinoma [35]. Another study showed that the knockdown of Linc-OIP5 suppressed the angiogenesis of the HUVECs through YAP1/Notch/NRP1 signalling in breast cancer [36]. In addition, we found that LINC00663 could positively regulate NR2F1 expression, and further experiments revealed that silencing LINC00663 repressed inflammation and vascular mimicry in BC cells by downregulating NR2F1. Moreover, RIP assay showed that EBF1 could bind LINC00663, and dual-luciferase assay and ChIP assay confirmed the binding site between EBF1 and NR2F1. Previously, overexpressed EBF1 rescued the inhibitive effect of upregulated TMPO-AS1 on BC development [37]. EBF1 was proved to be a target of LINC00261, and EBF1 overexpression could reverse the inhibition of LINC00261 on the proliferative and migration of thyroid cancer cells [38]. Consistently, our data also displayed that EBF1 overexpression reversed inhibitory effects of sh-LINC00663 on inflammation and vascular mimicry in BC cells, and further animal experiments showed that silencing LINC00663 decreased NR2F1 expression by binding EBF1, thereby repressing BC inflammation and angiogenesis.

In summary, our findings indicate that LINC00663 overexpression promotes inflammation and angiogenesis by binding EBF1 to increase NR2F1 expression, serving as a promising molecule for BC progression. Of course, there are still some shortcomings in this study. First, there is a relatively simple way to examine inflammation response in our cell and animal experiments, and multi-level *in vitro* and *in vivo* validations are needed in the follow-up study to strengthen the reliability of this conclusion. Second, there will be inevitable errors in our experimental process or data analysis, and more experiments and data will be needed to verify our results in the future. In any case, these findings offer a scientific basis for finding efficient immunotherapy targets for BC.

## Supplementary Material

S1.jpg

Supplementary Table 2.pdf

Supplementary Table 1.pdf

Supplementary Table 3.pdf

## Data Availability

The datasets used or analysed during the current study are available from the corresponding author on reasonable request.
