# Supplementary figures and images for: Silencing LINC00663 inhibits inflammation and angiogenesis through downregulation of NR2F1 via EBF1 in bladder cancer
Source: RNA Biol. 2024 Jun 18;21(1):9–22. doi: 10.1080/15476286.2024.2368304 (PMC11188801; doi:10.1080/15476286.2024.2368304)

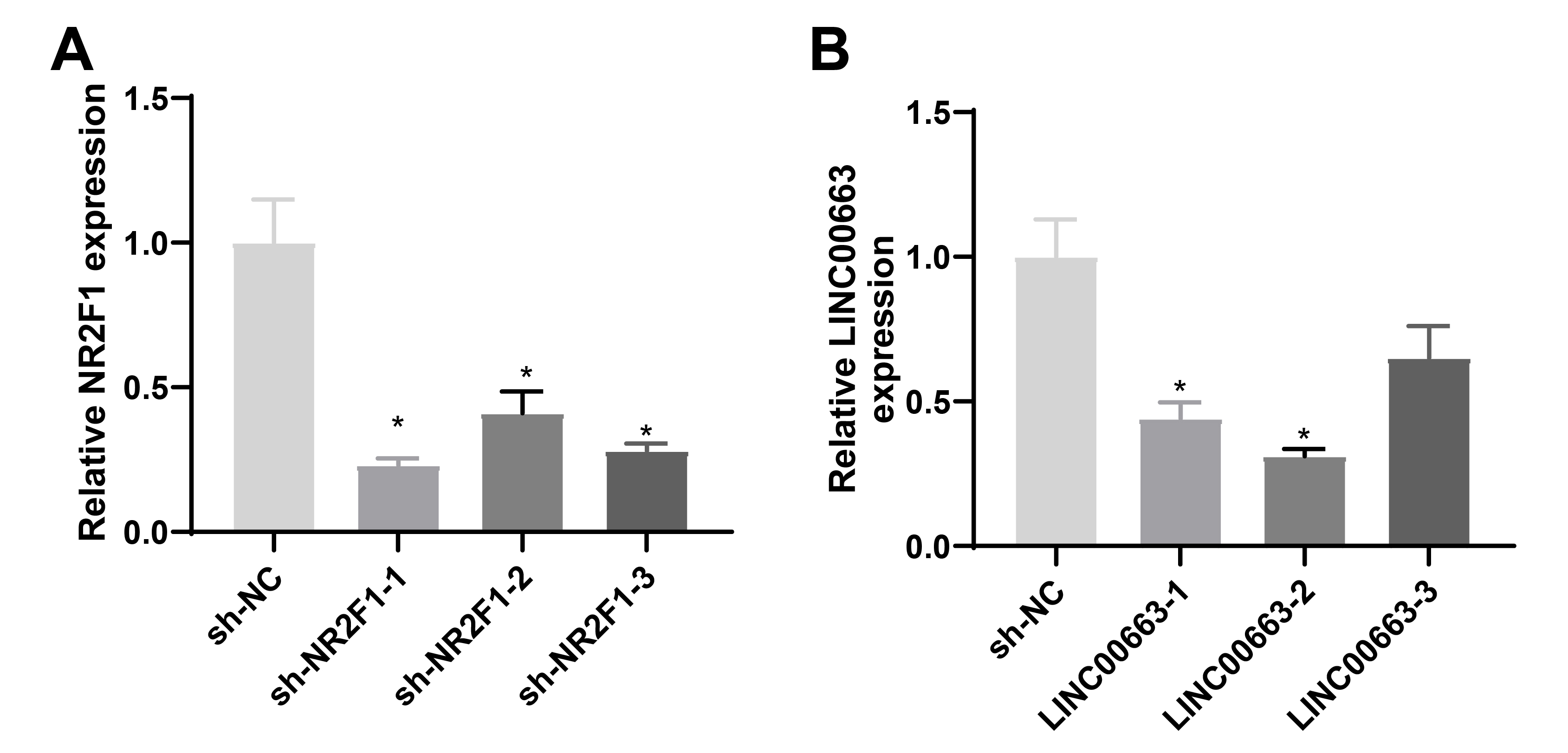

Supplement: S1.jpg [file KRNB_A_2368304_SM7351.jpg]
