# Supplementary material for: Silencing LINC00663 inhibits inflammation and angiogenesis through downregulation of NR2F1 via EBF1 in bladder cancer
Source: RNA Biol. 2024 Jun 18;21(1):9–22. doi: 10.1080/15476286.2024.2368304 (PMC11188801; doi:10.1080/15476286.2024.2368304)
Supplement: Supplementary Table 2.pdf [file KRNB_A_2368304_SM7350.pdf]

CancerCode	pvalue	HR	Lower	Upper	Hazard Ratio(95%CI)
TCGA-KIRP(N=27(	1.20E-03	1.310838	1.111788	1.545525	1.31(1.11,1.55)
TCGA-BLCA(N=39(	1.20E-03	1.173148	1.064695	1.292648	1.17(1.06,1.29)
TCGA-STAD(N=37(	0.01	1.152575	1.033137	1.285822	1.15(1.03,1.29)
TCGA-UCEC(N=16(	0.02	1.249248	1.029868	1.51536	1.25(1.03,1.52)
TCGA-STES(N=54(	0.06	1.090945	0.996586	1.194239	1.09(1.00,1.19)
TCGA-UVM(N=74)	0.06	1.279029	0.98464	1.661434	1.28(0.98,1.66)
TCGA-KIPAN(N=8(	0.11	1.0572	0.987981	1.131269	1.06(0.99,1.13)
TARGET-ALL-R(N(	0.13	1.062459	0.982103	1.14939	1.06(0.98,1.15)
TCGA-COADREAD(	0.16	1.121909	0.953796	1.319654	1.12(0.95,1.32)
TCGA-LIHC(N=34	0.16	1.080449	0.969653	1.203905	1.08(0.97,1.20)
TCGA-COAD(N=27(	0.2	1.123567	0.939378	1.343872	1.12(0.94,1.34)
TCGA-GBM(N=144)	0.24	1.135496	0.918226	1.404175	1.14(0.92,1.40)
TARGET-WT(N=80)	0.32	1.127972	0.887445	1.433691	1.13(0.89,1.43)
TCGA-BRCA(N=10(	0.42	1.039426	0.946155	1.141891	1.04(0.95,1.14)
TCGA-LUSC(N=46(	0.45	1.045517	0.932082	1.172757	1.05(0.93,1.17)
TCGA-THCA(N=50	0.47	1.157463	0.778036	1.721924	1.16(0.78,1.72)
TCGA-READ(N=90)	0.51	1.14245	0.764948	1.706248	1.14(0.76,1.71)
TCGA-SKCM-P(N=(	0.53	1.057453	0.889593	1.256987	1.06(0.89,1.26)
TCGA-OV(N=407)	0.54	1.024553	0.947579	1.10778	1.02(0.95,1.11)
TCGA-THYM(N=11(	0.89	1.033583	0.656775	1.626574	1.03(0.66,1.63)
TCGA-GBMLGG(N=(	4.10E-05	0.726784	0.624608	0.845674	0.73(0.62,0.85)
TCGA-KIRC(N=51(	6.10E-03	0.88816	0.815875	0.96685	0.89(0.82,0.97)
TCGA-HNSC(N=50(	0.06	0.920772	0.843946	1.004592	0.92(0.84,1.00)
TCGA-MESO(N=84)	0.13	0.886114	0.757494	1.036574	0.89(0.76,1.04)
TCGA-ACC(N=77)	0.15	0.835909	0.654674	1.067316	0.84(0.65,1.07)
TARGET-ALL(N=8(	0.21	0.941965	0.857858	1.034317	0.94(0.86,1.03)
TCGA-SKCM(N=44(	0.34	0.966873	0.902832	1.035458	0.97(0.90,1.04)
TARGET-NB(N=15	0.35	0.931705	0.803892	1.07984	0.93(0.80,1.08)
TCGA-CESC(N=27(	0.37	0.938363	0.816901	1.077884	0.94(0.82,1.08)
TCGA-LUAD(N=49(	0.42	0.952357	0.845592	1.072604	0.95(0.85,1.07)
TCGA-SKCM-M(N=(	0.49	0.97353	0.902887	1.0497	0.97(0.90,1.05)
TCGA-LGG(N=474)	0.59	0.93159	0.721071	1.20357	0.93(0.72,1.20)
TCGA-LAML(N=20(	0.72	0.991099	0.944236	1.040288	0.99(0.94,1.04)
TCGA-PCPG(N=17(	0.75	0.913207	0.518126	1.609545	0.91(0.52,1.61)
TCGA-SARC(N=25(	0.77	0.982814	0.875273	1.103568	0.98(0.88,1.10)
TCGA-PRAD(N=49(	0.79	0.928535	0.535633	1.609642	0.93(0.54,1.61)
TCGA-CHOL(N=33)	0.8	0.955411	0.676842	1.348633	0.96(0.68,1.35)
TCGA-UCS(N=55)	0.84	0.978566	0.796419	1.202372	0.98(0.80,1.20)
TARGET-LAML(N=	0.85	0.992888	0.920957	1.070438	0.99(0.92,1.07)
TCGA-TGCT(N=12(	0.88	0.96559	0.614154	1.518127	0.97(0.61,1.52)
TCGA-DLBC(N=44)	0.88	0.973965	0.697715	1.359593	0.97(0.70,1.36)
TCGA-KICH(N=64)	0.92	0.968823	0.540065	1.737972	0.97(0.54,1.74)
TCGA-ESCA(N=17(	0.96	0.994808	0.827507	1.195934	0.99(0.83,1.20)
TCGA-PAAD(N=17(	0.98	0.997871	0.851986	1.168736	1.00(0.85,1.17)
