# Supplementary material for: Silencing LINC00663 inhibits inflammation and angiogenesis through downregulation of NR2F1 via EBF1 in bladder cancer
Source: RNA Biol. 2024 Jun 18;21(1):9–22. doi: 10.1080/15476286.2024.2368304 (PMC11188801; doi:10.1080/15476286.2024.2368304)
Supplement: Supplementary Table 1.pdf [file KRNB_A_2368304_SM7349.pdf]

id	coef	HR	HR.95L	HR.95H	pvalue
NR2F1	0.212543	1.236819	1.024668	1.492896	0.026844
FXYD6	-0.22264	0.800404	0.607195	1.055092	0.114221
SORBS2	0.225529	1.252985	0.99301	1.581023	0.057322
DLGAP5	0.289528	1.335797	1.085638	1.6436	0.006208
PLIN4	-0.15122	0.859655	0.749217	0.986372	0.031119
ELN	0.198398	1.219448	1.018981	1.459354	0.030375
SERPINF1	0.148885	1.16054	1.004245	1.34116	0.043658
TMOD1	0.320967	1.378461	1.026357	1.851357	0.032938
ARHGEF25	0.177129	1.193785	0.948283	1.502845	0.13158
MRVI1	-0.35618	0.700346	0.500713	0.979571	0.037477
