# Supplementary material for: Silencing LINC00663 inhibits inflammation and angiogenesis through downregulation of NR2F1 via EBF1 in bladder cancer
Source: RNA Biol. 2024 Jun 18;21(1):9–22. doi: 10.1080/15476286.2024.2368304 (PMC11188801; doi:10.1080/15476286.2024.2368304)
Supplement: Supplementary Table 3.pdf [file KRNB_A_2368304_SM7348.pdf]

Cnacer Type	LncRNA ID	LncRNA Symbol	TF ID	TF Symbol
BLCA	ENSG00000266904	LINC00663	ENSG00000164330	EBF1

The Correlation coefficient in lncRN

Gene ID	Gene Symbol	LncRNA Type	Correlation coefficient in lncRNA low expression group
ENSG00000175745	NR2F1	pan	1.60E-01

A expression group

Correlation coefficient in lncRNA high expression group	Score	The Fisher transformation of correlation coefficient in lncRNA low expression grou
6.56E-01	9.99E-01	1.61E-01

The Fisher transformation of correlation coefficient in lncRNA high expression group	The probability in discovery set	The probability in validation set
7.86E-01	5.30E-01	7.47E-01
